# Establishing political priority for global mental health: a qualitative policy analysis

**DOI:** 10.1093/heapol/czac046

**Published:** 2022-06-13

**Authors:** Valentina Iemmi

**Affiliations:** Department of Health Policy, London School of Economics and Political Science, Houghton St., London WC2A 2AE, UK

**Keywords:** Political priority, global mental health, COVID-19, qualitative study

## Abstract

Mental disorders represent the leading cause of disability worldwide, yet they remain a low global health priority. This paper uses a case study methodology and different data sources (35 interviews and documents) to analyse factors that have shaped the generation of political priority for global mental health over the past three decades (1990–2020) and their changes over time. The Shiffman and Smith framework on determinants of political priority for global health issues is used to organize data into themes: actor power, ideas, political context and issue characteristics. Global mental health has gained political attention, especially over the past decade, yet support remains limited. Findings reveal that actor power is undermined by a fragmented policy community, the absence of one guiding institution or coordination mechanism and little civil society mobilization. Public portrayal of the issue is divided, hampered by the absence of a common understanding by the community and by stigma. Some policy windows have been missed and a strong global governance structure is lacking. Credible indicators and evidence on simple cost-effective solutions, especially in low- and middle-income countries, are scarce. However, opportunities are arising, including an increasing number of leaders and grassroots organizations, multiple arguments for action and integrated solutions resonating with broader audiences, widening political support at the national level, an emerging global governance structure and an expanding evidence base on the scale of the problem and available solutions. The results point to three technical and four political challenges that advocates need to address to increase political support over the next decade.

Key messages

- Global mental health has gained political attention, especially over the past decade, yet support remains limited.
- Advocates face several challenges, including a fragmented policy community, a divided public portrayal, lack of a strong global governance structure and few credible indicators.
- Opportunities are arising, including an increasing number of leaders and grassroots organizations, multiple arguments for action, widening political support and an expanding evidence base on cost-effective interventions.
- The results point to three technical and four political challenges that advocates need to address to increase political support over the next decade.

## Introduction

Mental disorders (including common and severe mental disorders, child behavioural disorders, neurodevelopmental disorders, substance use disorders, dementia and self-harm) represent the leading cause of disability worldwide and the third leading cause of global burden of disease after cardiovascular diseases and cancer (Global Burden of Disease Collaborative Network, 2020). Most of the burden is in low- and middle-income countries (LMICs) where 81% of people with mental disorders live (Global Burden of Disease Collaborative Network, 2020). Estimates are increasing due to demographic and epidemiological changes and worsening social determinants of mental health (e.g. inequalities) (Patel *et al.*, 2018), exacerbated by coronavirus disease 2019 (COVID-19), its policy responses and their socio-economic consequences (Santomauro *et al.*, 2021).

Despite the availability of cost-effective interventions (Chisholm *et al.*, 2016), mental disorders remain a low priority. Fewer than 20% of people living with mental disorders receive support (Patel *et al.*, 2018), and little funding is allocated to the issue: a global median of 2.1% of government health budget (WHO, 2021d) and 0.4% of development assistance for health (Charlson *et al.*, 2017). It is therefore important to understand the determinants of political priority for global mental health, and challenges and opportunities facing the policy community. However, evidence is limited.

A brief analysis of the prioritization of global mental health during the 2000s reveals that strong champions promoted the issue at the global level and the evidence on the scale of the burden and cost-effective solutions increased (Tomlinson and Lund, 2012). However, advocacy efforts were hindered by the lack of unity among the policy community, a unified message, a global governance structure and missed opportunities (Tomlinson and Lund, 2012). A study exploring the political prioritization of non-communicable diseases as a global health issue highlights the exclusion of mental disorders from the non-communicable diseases category until their inclusion in 2018 (Heller *et al.*, 2019). Over the past decade, political attention has increased as demonstrated by the inclusion of mental health in the Sustainable Development Goals (SDGs) (UN, 2015). COVID-19 has elevated the issue at an unprecedented scale, with the United Nations (UN) Secretariat and World Health Organization (WHO) World Health Assembly dedicating a policy brief and an agenda item to it, respectively (UN, 2020; WHO, 2021a). This paper analyses factors that have shaped the generation of political priority for global mental health over the past three decades preceding the COVID-19 pandemic (1990–2020) and their changes over time and identifies challenges and opportunities to inform discussion on the prioritization of global mental health over the next decade post-COVID-19. It builds upon a previous study (Tomlinson and Lund, 2012) expanding the scope to the last 10 years and disaggregating analyses by factor and decade.

## Methods

I used a case study methodology to produce in-depth analysis of the generation of political priorities for global mental health, triangulating different data sources (interviews and documents) to minimize bias (Gerring, 2006). Between February and December 2018, I conducted in-depth interviews with key informants working in international organizations in global health and experts in global mental health as part of a larger study (Iemmi, 2021). I selected participants using purposeful sampling and snowballing until saturation, stratified by sector (public, private, third sectors and multisector partnerships) to capture population heterogeneity. Informed consent was sought from participants before the interview in writing or orally. Interviews lasted on average 1 h, were conducted face-to-face and via telephone or Skype and digitally recorded when permitted. They comprehended a set of questions on factors that have shaped political priority for mental disorders as a global issue since 1990. Recordings were transcribed *verbatim*, alongside interview notes. In addition, I searched peer-reviewed and grey literature and policy documents in institutional websites of key international organizations and initiatives using key words for mental health and for determinants of political priority for global health initiatives as identified by Shiffman and Smith (2007) (Table 1, Supplementary Appendix 1). Documents were sourced in English, French, Italian, Portuguese and Spanish.

**Table 1.** Shiffman and Smith framework on determinants of political priority for global health initiatives (Shiffman and Smith, 2007)

Theme	Sub-theme	Definition
Actor power The strength of the individuals and organizations concerned with the issue	Policy community cohesion	The degree of coalescence among the network of individuals and organizations that are centrally involved with the issue at the global level
	Leadership	The presence of individuals capable of uniting the policy community and acknowledged as particularly strong champions for the cause
	Guiding institutions	The effectiveness of organizations or coordinating mechanisms with a mandate to lead the initiative
	Civil society mobilization	The extent to which grassroots organizations have mobilized to press international and national political authorities to address the issue at the global level
Ideas The ways in which those involved with the issue understand and portray it	Internal frame	The degree to which the policy community agrees on the definition of, causes of and solutions to the problem
	External frame	Public portrayals of the issue in ways that resonate with external audiences, especially the political leaders who control resources
Political contexts The environments in which actors operate	Policy windows	Political moments when global conditions align favourably for an issue, presenting opportunities for advocates to influence decision makers
	Global governance structure	The degree to which norms and institutions operating in a sector provide a platform for effective collective action
Issue characteristics Features of the problem	Credible indicators	Clear measures that show the severity of the problem and that can be used to monitor progress
	Severity	The size of the burden relative to other problems, as indicated by objective measures such as mortality levels
	Effective interventions	The extent to which proposed means of addressing the problem are clearly explained, cost-effective, backed by scientific evidence, simple to implement and inexpensive

I utilized the Shiffman and Smith framework to organize data into themes corresponding to determinants of political priority for global health initiatives (Shiffman and Smith, 2007). The framework includes 4 themes and 11 sub-themes: actor power (policy community cohesion, leadership, guiding institutions and civil society mobilization), ideas (internal frame and external frame), political contexts (policy windows and global governance structure) and issue characteristics (severity, effective interventions and credible indicators) (Table 1). The unit of analysis was the individual or manuscript. I triangulated data across sources to enhance robustness and minimize bias. Analyses of interviews and interview notes were performed in NVivo12, and interview quotations were anonymized to ensure confidentiality. To ensure factual accuracy, feedback from six participants on the results draft was included. Ethical approval was obtained from the London School of Economics and Political Science (LSE) Research Ethics Committee (Reference No. 000589).

## Findings

Thirty-five participants from 12 countries in three regions (Africa, the Americas and Europe) and two LMICs were interviewed (Supplementary Appendix 2). Most individuals who have worked in global mental health over the past three decades come from high-income countries where most of the interviewees were based. Results show that global mental health has gained political attention, especially over the past decade, yet support remains limited. Over the past three decades, all factors analysed became more favourable except one for which change was negligible: guiding institutions (Table 2). This section summarizes the results for each theme and sub-theme (see Supplementary Appendix 4 for a summary table). Illustrative quotations are reported in Supplementary Appendix 3.

**Table 2.** Global attention to mental health in 2000, 2010 and 2020

	2000	2010	2020
Actor power			
Policy community cohesion	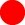	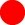	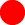
Leadership	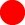	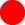	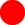
Guiding institutions	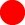	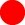	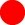
Civil society mobilization	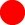	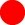	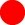
Ideas			
Internal frame	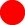	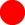	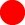
External frame	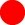	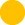	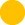
Political contexts			
Policy windows	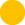	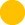	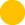
Global governance structure	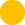	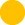	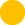
Issue characteristics			
Credible indicators	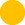	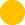	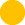
Severity	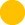	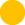	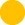
Effective interventions	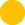	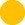	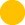

Three circles illustrate favourability of each determinant of political priority for mental health in 2000, 2010 and 2020, respectively. Favourability corresponds to the number of barriers to each determinant. Red, many; orange, few; green, none.

### Actor power

#### Policy community cohesion

Policy communities are networks of individuals and organizations concerned by an issue and operating at a global level: their coalescence is fundamental to the success of initiatives. The global mental health policy community has grown over the past three decades, reflecting the proliferation of actors involved in the issue worldwide (Iemmi, 2019). However, the policy community remains fragmented due to unsolved tensions among actors with different interests and approaches (Iemmi, 2021) (interviews 8, 16 and 32). Since the 1990s, tensions persist vis-à-vis the private sector, especially the pharmaceutical industry, based on concerns about profit prioritization (Fava, 2007), and vis-à-vis the radical critics of biomedical psychiatry contesting the misuse of high-income countries’ care models in LMICs (Summerfield, 2008) (interviews 1, 5, 8, 16, 17 and 32). Since the Convention on the Rights of Persons with Disabilities (CRPD) entered into force in 2008 (UN, 2007), similar frictions linger with the disability community contending right-based approaches in mental health (Patel *et al.*, 2018) (interview 19).

Opportunities for coalescence have been offered by the establishment of global networks of individuals and organizations promoting the issue. Since 2008, the Movement for Global Mental Health (MGMH) has attempted to strengthen the policy community with limited results partly attributable to the limited representation of local understanding of the issue and its solutions and the limited mobilization of local activists (Patel *et al.*, 2011; Howell *et al.*, 2017; Campbell, 2020; MGMH, 2021). More recently, the Global Mental Health Action Network (GMHAN) has offered a platform for collaborations across actors (GMHAN, 2021) and an organization aiming to unite the field has been established (United for Global Mental Health, UGMH) (UGMH, 2021). Similarly, while tensions with the private sector persist in some areas (e.g. pharmaceutical industry), they have been appeased by the increasing engagement of private actors in less contentious mental health activities such as philanthropy, technology and workplace support (Fu *et al.*, 2020; Iemmi, 2020; Global Business Collaboration for Better Workplace Mental Health, 2021).

#### Leadership

Leaders are strong advocates for an issue, able to coalesce and provide direction to the policy community. Few in the past, strong champions capable to unite the global mental health policy community are increasing in number and breadth, including influential individuals organized in informal networks, high-level leaders in international organizations and governments, and celebrities. Authoritative and charismatic individuals mainly from academia, often key figures in informal networks, were initially crucial in generating evidence and contributing to the work of key international institutions (e.g. WHO) (Gulbinat *et al.*, 2004; The Lancet, 2007; 2011; Patel *et al.*, 2018) (interviews 8–11, 16–19 and 32). During the 1990s and early 2000s, key members of the International Consortium on Mental Health Policy and Services advanced global mental health by strengthening evidence on the mental health system in LMICs and advocating for the first mental health position at the World Bank (Whiteford, 1999; Gulbinat *et al.*, 2004) (interviews 7 and 32). Since the mid-2000s, key individuals mainly from academia and part of another informal network have contributed to elevate global mental health through high-level publications (The Lancet, 2007; 2011; Patel *et al.*, 2018) and collaborations with international organizations (WHO, 2010c; WHO & Mental Health and Poverty Project 2010) (interviews 8–11, 16 and 18). Yet, the focus on academia had limits.

Exceptional in the past, a growing (yet limited) number of high-level leaders in international organizations have increasingly promoted the issue, especially over the past decade (interviews 10, 23 and 32). Gro Harlem Brundtland was the first WHO Director-General who gave an important visibility to mental health through the first World Health Report, World Health Day and dedicated seminars during the World Health Assembly in 2001 (WHO, 2001b). More recently, heads of key UN agencies have heightened mental health. The UN Secretary-General António Guterres has launched a UN system-wide strategy on mental health and well-being (2018–2023) and has supported a policy brief (UN, 2018b; 2020). The WHO Director-General Tedros Adhanom Ghebreyesus has launched a WHO Special Initiative for Mental Health (2019–2023) to accelerate mental health implementation in 12 priority countries and has supported numerous activities (WHO, 2019; 2020a). The former United Nations Children’s Fund (UNICEF) Executive Director Henrietta Fore has prioritized mental health support of children and adolescents in the agency activities (UNICEF, 2019a; 2021).

Few government leaders and champions have contributed to the advancement of mental health as a global issue. During the 1990s, former US First Lady Rosalynn Carter with members of the Committee of International Women Leaders for Mental Health advocated for mental health worldwide (Carter, 1997). In 2013, the UK Prime Minister David Cameron convened the G8 Dementia Summit to accelerate global action against dementia (DHSC, Prime Minister’s Office, 2013) (interviews 10 and 32). Finally, over the past decade, an increasing number of celebrities have raised mental health worldwide, such as Lady Gaga (Gaga and Adhanom, 2018) (interview 23).

#### Guiding institutions

Guiding institutions are organizations or coordinating mechanisms mandated to guide an initiative: often starting as informal networks, their formalization is critical to the initiative’s survival. The absence of a single guiding institution or coordination mechanism has hampered the coordination of efforts to elevate mental health at the global level. However, multiple institutions have held prominent roles, including international and civil society organizations. WHO has occupied a sustained and increasingly privileged position, by strengthening evidence, awareness and political support, and establishing global plans for action. Over the past three decades, it has expanded considerably its activities on the issue. During the 1990s, it launched *Nations for Mental Health*, an initiative to raise the profile of mental health worldwide (WHO, 2002), and the WHO World Mental Health Survey Initiative in collaboration with Harvard University to catalyse the collection of epidemiological data worldwide (Kessler and Üstün, 2004) (interview 22). During the 2000s, WHO promoted mental health as a public health issue (WHO, 2001b), launched the Mental Health Gap Action Programme (mhGAP) to provide guidelines for the treatment of mental disorders in low-resource settings (WHO, 2008; 2010c; 2015b; 2015c; 2016) and produced resources for decision makers such as mental health atlases, mental health country profiles and guidelines for mental health policy and service development (WHO, 2001a; 2005; 2011; 2015a; 2018a; 2021d; 2021f; 2021g).

Over the past decade, WHO established global plans for action for mental health [Comprehensive Mental Health Action Plan 2013–2020 (MHAP), recently updated and extended to 2030] (WHO, 2013d; 2021c) and single conditions (alcohol use and dementia) (WHO, 2010b; 2017b), strengthened awareness and political support through dedicated campaigns (e.g. 2017 World Health Day campaign on depression) (WHO, 2021h) and high-level meetings (e.g. 2016 WHO-World Bank meeting on mental health) (Mnookin *et al.*, 2016) and expanded evidence for decision makers (e.g. mhGAP guidelines and QualityRights initiative) (WHO, 2012; 2015b; 2016; Funk and Drew, 2017) (interviews 21, 31 and 33). In addition, since the 2010s, an increasing number of UN agencies have promoted the issue often in collaboration with WHO, such as UNICEF and UN High Commissioner for Refugees (UNHCR) (UNHCR, 2021; UNICEF, 2019b; 2020; WHO, UNICEF, 2021) (interview 28).

Often in formal or informal collaborations with WHO and other UN agencies, key civil society organizations have played an important (yet limited) role in leading global advocacy for mental health, including non-governmental organizations and lived experience groups. Among non-governmental organizations, since its establishment in 1948 the World Federation for Mental Health (WFMH) has raised the issue at the global level through its consultative status with the UN Economic and Social Council and collaborations with WHO (e.g. World Mental Health Day) (WFMH, 2021), and since 2018 UGMH has contributed to global advocacy (Saxena and Kline, 2021). Among lived experience groups, the World Network of Users and Survivors of Psychiatry (WNUSP) between the 1990s and the 2000s and the Global Mental Health Peer Network (GMHPN) since 2018 have advocated for the rights of people with lived experience of mental disorders worldwide (GMHPN, 2021; WNUSP, 2021).

#### Civil society mobilization

Grassroots organizations are critical for pressuring international and national political authorities to address an issue at the global level. The mobilization of civil society organizations working in global mental health has intensified over the past two decades. While during the 1990s, non-governmental organizations (e.g. WFMH) and lived experience groups (e.g. WNUSP) were limited and unable to put pressure on international and national political authorities, their efforts strengthened during the 2000s with the arrival of new organizations focusing on (e.g. BasicNeeds) or expanding their remit to include mental health (e.g. Christian Blind Mission, CBM) (CBM, 2021) (interviews 21, 28 and 32). The proliferation of actors over the past decade brought new advocacy (e.g. UGMH) and lived experience (e.g. GMHPN) groups intensifying pressure on political authorities at the national and global levels (Iemmi, 2019). In addition, this has benefitted from growing support from civil society organizations advocating for other issues increasingly integrating mental health components (e.g. diabetes) (Sartorius and Cimino, 2012) and from the recent establishment of a global partnership of national campaigning groups (Speak Your Mind) (Speak Your Mind, 2021) (interview 32). Yet, strong civil society organizations, especially lived experience and family groups, remain limited at both national and global levels compared with other health conditions (e.g. human immunodeficiency virus infection and acquired immunodeficiency syndrome, HIV/AIDS) (Campbell, 2020) (interviews 1, 10, 18, 19 and 21).

### Ideas

#### Internal frame

The common understanding of an issue and its solutions by a policy community is fundamental to its coalescence. The policy community is slowly converging on a common understanding of mental disorders, as a neglected issue including a heterogeneous group of conditions caused by bio-psycho-social factors, affecting different periods and dimensions of the life of individuals and their families/carers, hence requiring multisectoral and life-course approaches (Patel *et al.*, 2018). Solutions are increasingly advanced beyond targeted healthcare interventions and integrated across other health conditions (e.g. HIV/AIDS), sectors (e.g. education, employment and criminal justice) and issues (e.g. gender, disability and youth) with an amplifier effect (Iemmi, 2021). However, disagreements persist. Definitions often include some neurological disorders reflecting previous conceptualizations and practices (Vigo et al., 2016; WHO, 2001b; 2008; World Bank, 1993) and marginalize local understanding (Summerfield, 2008), diverse terminologies (e.g. mental health, mental disorders, psychosocial disabilities and mental well-being) reinforce ambiguity (IASC, 2007; Layard et al., 2013), and divergent solutions are foregrounded by different actors (interview 22).

During the 1990s, mental disorders were predominantly understood through two models: the biomedical model supporting standalone interventions often restricted to individual-level healthcare and the cultural model advancing culturally appropriate community-level solutions (Kleinman, 1987). Two models became prominent in the 2000s: the bio-psycho-social model foregrounding multiple biological, psychological and social factors to be addressed through evidence-based solutions in healthcare and beyond (e.g. WHO mhGAP guidelines) (WHO, 2001b; 2010c) and the human rights model promoting human rights-based approaches (e.g. community-based rehabilitation) (UN, 2007; WHO, 2010a). The growing focus on happiness and well-being and social determinants of health (especially poverty) over the past decade has facilitated a further expansion of the understanding of the issue to include positive aspects and of solutions integrated across sectors and over the lifetime (Layard *et al.*, 2013; Lund *et al.*, 2018) (interviews 6, 8, 10, 15, 17, 28, 29, 32 and 34). The latter has benefitted from the growing interest in horizontal issues, such as non-communicable diseases and disability, requiring multisectoral and life-course approaches (interviews 6, 10, 15, 28 and 34).

#### External frame

Different issues are portrayed using different frames (e.g. public health and economic) which resonates with different external audiences: the use of coherent and appealing frames is key to the success of initiatives (Goffman, 1974). Failure to solve disagreements within the global mental health community has hindered the generation of a coherent public portrayal of the issue (interview 23). However, the development of multiple arguments for action and integrated solutions have resonated with broader audiences and attracted increased political attention (Iemmi, 2021). Since the 1990s, two reasons for action have been used: as a public health measure to address the gap between the size of the problem and available support and as a human rights issue to tackle abuses, such as chaining, torture and sterilization, and lack of parity between mental and physical health (UNHRC, 2019). They have sometimes been combined into a moral argument for action, used to create a sense of urgency and emotional connections (Patel *et al.*, 2006; Patel and Farmer, 2020) (interviews 3, 8, 10, 12 and 32). Since the 2000s, the economic argument has been increasingly used to emphasize losses in productivity in individuals with mental disorders and their families/carers alongside losses in the gross domestic product (WHO, 2003; 2013c; Saxena *et al.*, 2007; Bloom *et al.*, 2011). Over the past decade, mental disorders have also been presented as a development issue (Patel *et al.*, 2018), a cost-effective investment (Chisholm *et al.*, 2016) and a major cause of unhappiness (Layard *et al.*, 2013) (interview 4).

Although decreased stigma has benefitted advocacy by exposing the substantial size of the problem and increasing audience receptivity, it continues to hamper public and political support (McGinty *et al.*, 2016) (interviews 18 and 26). This reduction has been facilitated by deinstitutionalization and integration of people living with mental disorders in the community, awareness and anti-stigma campaigns at the national and global levels (UNDESA, 2014; Thornicroft *et al.*, 2016; WHO, 2021h), and improved media portrayal promoted by media training and guidelines (The Carter Center, 2015; 2021; Rice-Oxley, 2019) (interviews 1, 4, 6, 19 and 23).

### Political contexts

#### Policy window

Policy windows represent moments in time when global conditions are favourable for an issue and policy communities have strong opportunities to influence decision makers (e.g. humanitarian disasters and political fora). Global mental health advocates have increasingly seized policy windows, which have grown over the past decade (interviews 5, 17, 26 and 31). After failing to link the issue to the Millennium Development Goals (Miranda and Patel, 2005), they successfully secured its inclusion into the SDGs under the health goal and across other goals through the leave-no-one-behind agenda (UN, 2015). They have increasingly collaborated with key international organizations and governments on high-level meetings dedicated to the issue which have galvanized the community, yet attracted little political attention unless convened by governments ready to make commitments (interviews 8, 10, 16, 17, 21, 31, 33 and 34).

For instance, while tangible commitments were not visible after the 2016 WHO and World Bank meeting on global mental health (Mnookin *et al.*, 2016), the 2013 G8 Dementia Summit convened by the UK government led to increased political attention, funding and global response for dementia (WHO, 2017a; 2017b; DDF, 2021). Similarly, the Global Ministerial Mental Health Summits convened by the UK government in 2018 and The Netherlands in 2019 resulted in some political commitments (Department of Health and Social Care, 2018; Dutch Ministry of Foreign Affairs, 2019) (interviews 10, 16, 17, 21, 31 and 33). However, disagreements and lack of coordination among global mental health actors have hampered efforts, such as independent advocacy to promote the inclusion of mental health in the SDGs by different groups (Kuriansky, 2016; Votruba and Thornicroft, 2016).

More recently, other policy communities have elevated mental health in broader high-level meetings. These have increasingly integrated mental health components into the issue they are promoting, such as non-communicable diseases (e.g. third UN high-level meeting on non-communicable diseases in 2018) (UN General Assembly, 2018) and disability (e.g. Global Disability Summit in 2018) (DFID, 2021) (interviews 13, 16 and 31).

The policy community has failed to capitalize on humanitarian emergencies, with some exceptions. The 2004 tsunamis in Banda Aceh, Indonesia and Sri Lanka marked a turning point used to widen understanding and action in mental health in humanitarian settings (IASC, 2007; WHO, 2013a) (interviews 19, 22 and 24). The current COVID-19 pandemic is being leveraged to increase attention on the issue, yet it is too early to gauge its impact. However, building on an increasing interest from governments (e.g. The Netherlands), mental health response during and after humanitarian emergencies has gained traction over the past few years (Dutch Ministry of Foreign Affairs, 2019). In addition, global advocacy has benefitted from an expanding (yet limited) public and political support for the issue at the national (WHO, 2019) and regional (e.g. Asian-Pacific Economic Cooperation, APEC) (APEC, 2014) levels over the past decade (interviews 4, 5, 7, 8, 17–19, 26 and 33).

Advocates have successfully produced key publications to propel the issue further. For instance, the 1995 Harvard report promoted mental health as a global issue capturing the attention of the UN Secretary-General Boutros Boutros-Ghali and influencing WHO activities (Desjarlais *et al.*, 1995; WHO, 2002). The first and second Lancet Series on Global Mental Health and the *Lancet* Commission on global mental health and sustainable development brought the authority of the journal behind the issue (The Lancet, 2007; 2011; Patel *et al.*, 2018) (interviews 4, 7, 17, 22 and 25).

#### Global governance structure

The global governance structure consists in the set of norms (e.g. international legislations) vis-à-vis an issue and the institutions negotiating and enforcing them, which provide a platform for collective action. Over the past two decades, the emergence of a global governance structure has increasingly supported collective action for mental health (interviews 4, 7, 10–12, 18, 19, 22, 24 and 31). Global conventions, frameworks and plans have increased (Tsutsumi *et al.*, 2015). Building upon previous instruments (e.g. the 1971 declaration on the rights of mentally retarded persons) (UN General Assembly, 1971), in 1991 the UN General Assembly adopted key principles for the protection and support of persons with mental illness (UN General Assembly, 1991). Similarly reinforcing previous instruments (e.g. the 1975 declaration on the rights of disabled persons) (UN General Assembly, 1975), the CRPD came into force in 2008 and provided an international legal instrument that strengthened the human rights argument and spurred action at the global level (e.g. WHO QualityRights programme) (UN, 2007; Funk and Drew, 2017).

During the 2010s, three political moments represented important milestones (interviews 4, 7, 10, 12, 18, 19, 24 and 31). The WHO Comprehensive MHAP set objectives, targets and recommended actions to improve mental health worldwide (WHO, 2013d). The inclusion of mental health in the SDGs offered a global framework for action (UN, 2015). Building on the Hyogo Framework for Action 2005–2015 (UNISDR, 2005), the Sendai Framework for Disaster Risk Reduction 2015–2030 foregrounded psychosocial support during, before and after humanitarian disasters (UNISDR, 2015). However, the impact of global conventions, frameworks and plans has been hampered by the lack of enforcement mechanisms and country prioritization, creating a disconnect between global discourse and local realities (Eaton *et al.*, 2021) (interviews 11, 19 and 22).

Normative shifts over the past two decades have influenced the global mental health governance structure favourably: an increasing focus on horizontal issues such as disability and non-communicable diseases (UN, 2007; 2018a; WHO, 2018b) and the inclusion in global decision-making of youth, promoting action to address mental disorders, one of the leading causes of illness, disability and death among them (e.g. 2021 G7 Youth Summit, Y7) (Y7, 2021) (interviews 1, 3, 6, 13 and 16). In particular, WHO’s inclusion of mental health in non-communicable diseases in 2018 widened relevant global plans (WHO Global Action Plan for the prevention and control of non-communicable diseases 2013–2020, recently extended to 2030) (WHO, 2013b; 2021b) and instruments (e.g. UN Inter-Agency Task Force on NCDs, UNIATF) (UNIATF, 2021). Coordinated action has profited from stronger institutions and the establishment of coordinating groups dedicated to the issue (e.g. Inter-Agency Standing Committee reference group on mental health and psychosocial support in emergency settings in 2007; International Alliance of Mental Health Research Funders in 2010) (IASC Reference Group on Mental Health and Psychosocial Support in Emergency Settings, 2021; IAMHRF, 2021b) (interviews 18, 29 and 34).

### Issue characteristics

#### Credible indicators

Issues with credible indicators are more likely to attract political attention due to the ability to capture the scale of the problem and to monitor progress. The scarcity of credible indicators for mental health due to issue characteristics (e.g. few biomarkers, relapses and heterogeneous group of conditions) and lack of consensus has made tractability difficult, hampering comparison with other health conditions and political support (Wolpert, 2018) (interviews 4, 16, 20 and 28). At the population level, epidemiological and system indicators have improved over the past two decades through global initiatives (e.g. WHO World Mental Health Survey Initiative and WHO Mental Health Atlas) (Kessler and Üstün, 2004; WHO, 2021d) and have been included among the SDGs indicators (e.g. suicide mortality rate) (UN General Assembly, 2017), yet methodological challenges remain (e.g. undercounting) (Snowdown and Choi, 2020). At the individual level, no meaningful improvement in indicators has led to the use of proxies (e.g. school/work absences and psychiatrist visits) during evaluations.

#### Severity

Issues representing larger size of the burden relative to others are more likely to be prioritized as perceived more serious by decision makers. The epidemiological and economic burden of mental disorders has increased worldwide over the past three decades (Bloom *et al.*, 2011; Whiteford *et al.*, 2013; 2015), partly due to demographic and epidemiological transitions (e.g. increasing population in age groups more likely to experience mental disorders and decreasing communicable disorders) and increase in stressors (e.g. inequalities) (Lund *et al.*, 2018; Patel *et al.*, 2018) (interview 16). Since the 1990s, mental disorders have represented a substantial and growing portion of the global burden of disease, mainly driven by disability (Patel *et al.*, 2018). The economic burden of mental disorders is the largest across non-communicable diseases, estimated at US$2.5 trillion in 2010, and expected to raise to US$6.1 trillion in 2030, with about two-thirds attributable to productivity losses (Bloom *et al.*, 2011). Those figures increase to US$8.5 and US$16.1 trillion, respectively, when capturing the intrinsic value of suffering and life (Bloom *et al.*, 2011).

Despite improvements, paucity of robust and objective data has hindered advocacy. Scarce in the 1990s (World Bank, 1993), epidemiological data have increased through extended global efforts in the 2000s (e.g. WHO World Mental Health Survey Initiative) and the collection of subnational-level data over the past decade (Kessler and Üstün, 2004; Kessler and Ustun, 2008; India State-Level Disease Burden Initiative Mental Disorders Collaborators, 2020). Few routinely collected indicators by periodic surveys and routine health information systems have meant that global figures have relayed heavily on estimations based on complex epidemiological models, especially for LMICs (GBD 2019 Diseases and Injuries Collaborators, 2020) (interviews 18 and 22). Similarly, while the availability of financial data has increased, the advancement of integrated solutions has challenged their accuracy (Charlson *et al.*, 2017; WHO, 2018a; Woelbert *et al.*, 2021) (interviews 20 and 22).

#### Effective interventions

Issues with evidence-based simple and cost-effective solutions are easier to promote as more appealing to decision makers in charge of resources. The evidence base on cost-effective and often low-cost mental health interventions has been growing extensively over the past three decades (Patel *et al.*, 2016) (interviews 16, 21, 26 and 29). Scanty and mainly limited to high-income countries in the 1990s, it expanded during the 2000s with the first clinical trials in LMICs allowing to demonstrate the feasibility of mental health interventions in low-resource settings (Araya *et al.*, 2003; Bolton *et al.*, 2003; Patel *et al.*, 2003). The evidence augmented further over the past decade, with intensifying economic evaluations and systematic reviews and meta-analyses (Chisholm *et al.*, 2016; CGMH, 2021; Cochrane Mental Health and Neuroscience, 2021).

However, challenges persist (interviews 4, 8, 11, 12, 14, 16, 17, 19, 21, 26 and 28). While dissemination has benefitted from the use of policy reports, clinical guidelines and knowledge brokers (e.g. Mental Health Innovation Network) (WHO, 2010c; MHIN, 2021), the issue remains misperceived as a novel field. Its multisectoral and life-course features mean that solutions are often complex and integrated, lacking the attractiveness of simple low-cost interventions (e.g. vaccinations). Finally, while increasing, evidence on solutions beyond healthcare and evidence on implementation and scalability across different settings are limited, especially in LMICs (Chisholm *et al.*, 2016; Gunnell *et al.*, 2017; Alves *et al.*, 2019).

## Discussion

Global mental health has gained political attention, especially over the past decade (Table 2, Supplementary Appendix 4), yet it faces several challenges across the four groups of factors shaping political support (Table 3). The main barriers undermining actor power are a fragmented policy community, few leaders especially outside academia, absence of one guiding institution or coordination mechanism and little civil society mobilization. The findings align with a previous study that identified the lack of unity in the policy community as an important barrier to political attention to mental health in the 2000s (Tomlinson and Lund, 2012), and they show little improvement over the past decade. They reflect the experience of other global health initiatives, such as non-communicable diseases and maternal health, which faced challenges in actor cohesion, mobilization and leadership before attracting more political attention (Shiffman and Smith, 2007; Jönsson, 2014).

**Table 3.** Challenges and opportunities for increasing global attention to mental health

	Challenges	Opportunities
Actor power		
Policy community cohesion	Fragmented policy community due to tensions across actors, especially private sector actors	Proliferation of actors; growing global networks of individuals and organizations; establishment of an organization aiming to unite the field; increasing role of the private sector in less contentious activities
Leadership	Few unifying leaders, mainly from academia	Increasing number of high-level leaders and champions promoting the issue, especially outside academia
Guiding institutions	Absence of one guiding institution or coordination mechanism	Strengthening of the role of prominent organizations
Civil society mobilization	Little civil society mobilization at the global level, especially lived experience and family groups	Growing mobilization of grassroots organizations pressing international and national political authorities; increasing support from other policy communities advocating for issues integrating mental health components; establishment of global networks of grassroots organizations
Ideas		
Internal frame	No consensus on the definition of, causes of and solutions for the issue	Progressive convergence on a common understanding of the issue
External frame	No unified public portrayal; inconsistent terminologies; stigma	Development of several arguments for action and integrated solutions resonating with broader audiences; decreasing stigma
Political contexts		
Policy windows	Limited and missed policy windows	Growing numbers of high-level meetings focusing on or including mental health; COVID-19 pandemic bringing attention to the issue worldwide; widening public and political support at the national and regional levels
Global governance structure	Little global governance structure; no accountability mechanisms	Emerging global governance structure, including an international treaty (CRPD), global frameworks (SDGs and Sendai Framework) and a plan (MHAP); favourable normative shifts; rise of coordinating groups
Issue characteristics		
Credible indicators	Few credible indicators, mainly at the population level	Inclusion of mental health indicators into the SDGs and MHAP; launch of a global monitoring mechanism (CGMH 2030); establishment of an initiative to identify common mental health indicators at the individual level
Severity	Paucity of robust data, especially in LMICs	Increasing burden relative to other conditions; improving data quality
Effective interventions	Limited evidence on simple cost-effective and scalable solutions, especially in LMICs; misperception of the issue as a new field	Expanding evidence base, especially on integrated solutions; publication of a list of cost-effective mental health interventions; establishment of an initiative aiming to build country-level investment cases

CGMH 2013, Countdown Global Mental Health 2030; COVID-19, coronavirus disease 2019; CRPD, Convention on the Rights of Persons with Disabilities; LMICs, low- and middle-income countries; MHAP, Mental Health Action Plan 2013–2020; SDGs, Sustainable Development Goals; Sendai Framework, Sendai Framework for Disaster Risk

Additional barriers are posed by the absence of a common understanding and public portrayal of the issue by the policy community, aggravated by stigma. The results are consistent with the evidence for the 2000s (Tomlinson and Lund, 2012), yet they reveal amelioration over the past decade. The prominent role of public portrayal to gain political attention aligns with a large literature on framing in global health (McInnes *et al.*, 2012). The challenge posed by stigma reflects the experience of other stigmatized issues, such as abortion (Daire *et al.*, 2018).

With respect to political contexts, the main obstacles are missed policy windows and absence of a strong global governance structure. Although increasing collaborations with other policy communities has widened policy windows, competition and negotiation among actors hamper efforts, as experienced by other policy communities, for instance, collaborations on the Global Strategy for Women’s and Children’s Health by the maternal, newborn and child health and the sexual and reproductive health and rights policy communities (McDougall, 2016). The poor global governance structure in the 2000s concurs with the literature (Tomlinson and Lund, 2012), yet results unveil its strengthening over the past decade. Global mental health governance faces common challenges in global health: sovereignty restricting mental health to a national responsibility, multisectoral features of the issue poorly addressed in non-health arenas (e.g. education) and limited accountability of multilateral organizations and non-state actors (Frenk and Moon, 2013).

Regarding issue characteristics, the main deterrents are the scarcity of credible indicators and paucity of evidence on simple cost-effective solutions, especially in LMICs, worsened by the misperception of the field novelty. Although the scarcity of metrics and simple solutions align with evidence for the 2000s (Tomlinson and Lund, 2012), caution is required. The selection of indicators risks to narrow the policy agenda by focusing on specific dimensions (e.g. mortality) (Storeng and Béhague, 2014) and the adoption of simple solutions to universalize context-specific issues (Howell *et al.*, 2017). The generation of political priority for other global health issues, such as non-communicable diseases and newborn survival, has been hindered by similar difficulties, such as fragmented community, public portrayal and global governance structure, with slow progress over decades (Shiffman, 2010; Heller *et al.*, 2019).

However, opportunities are arising (Table 3). Mental health actors are growing in number and power: an organization aims to unite the community, strong champions are growing outside academia, prominent institutions strengthening and mobilization of grassroots organizations increasing. Different arguments for action and integrated solutions are resonating with broader audiences, favoured by decreasing stigma. Political contexts are becoming more favourable: increasing high-level meetings on or including mental health, COVID-19 bringing attention to the issue worldwide, conducive normative shifts and an emerging global governance structure. Finally, the evidence base on the burden of and cost-effective interventions for mental disorders is expanding.

This study provides a granular analysis of factors shaping the political prioritization of global mental health over the past three decades. However, it has limitations. The use of qualitative methods might raise concerns regarding the robustness of results and possible bias, which was mitigated by triangulation across different sources of data. The selection of participants using purposeful sampling might have led to selection bias, in particular vis-à-vis geographies, which was partly reduced by stratification by sector. The underrepresentation of participants from LMICs might have led to exclusion of different understanding of mental health and its prioritization, which was partly attenuated by the analysis of documents including some regional information (e.g. regional reports) and published in five languages used in the main LMICs. This study—in particular, data collection and analysis—was influenced by my positionality as a female researcher with over 10 years of experience in mental health policy and practice research, a non-native English speaker and affiliated with a university in a high-income country.

## Conclusion

The results point to three technical and four political challenges the global mental health community needs to address to gain political attention. The main technical challenges are few credible indicators, scarcity of robust data and limited evidence on implementation and scaling-up of solutions. The community might leverage existing initiatives aiming to determine common mental health metrics (IAMHRF, 2021a), to include mental health indicators in routine surveys (UNICEF, 2021), to identify cost-effective mental health interventions (WHO, 2021e) and to build country-level investment cases (WHO & United Nations Development Programme 2021).

The first political challenge is coalescing the policy community around a common request. Formal and informal groups (e.g. GMHAN) and a unifying organization (UGMH) might offer privileged platforms to solve disagreements and foster collaborations. Second is reaching a consensus upon an external frame resonating with political leaders using consistent terminology while continuing to address stigma. The community might build on available definitions (Patel *et al.*, 2018) and arguments (Iemmi, 2021) to articulate a common nuanced narrative that could be adapted to different audiences. In addition, it might systematically leverage other policy communities capitalizing on the integration of mental health across other health conditions, sectors and issues (Lund *et al.*, 2018; Collins *et al.*, 2020).

Third is creating stronger and long-lasting guiding institutions or coordination mechanism to lead and coordinate the initiative over time. The establishment of a sustainable partnership for global mental health might foster collective action (Vigo *et al.*, 2019), which could leverage global frameworks and plans (e.g. SDGs and MHAP) and the recently launched accountability mechanism to monitor mental health progress worldwide (Countdown Global Mental Health 2030) (Saxena and Kline, 2021). Collaboration with guiding institutions for other relevant issues is crucial to foster synergies, coordinating messages and actions (e.g. non-communicable diseases, communicable diseases and universal health coverage). Fourth is strengthening grassroots organizations at the global level and establishing more robust and durable connections with those at the national level. Existing global networks of national campaigning groups (e.g. Speak Your Mind) and people with lived experience (e.g. GMHPN) might facilitate links with national initiatives.

Further research could provide additional insights into the political prioritization process in global mental health. Qualitative studies could explore prioritization at the global level for different population groups (e.g. age groups) and for different mental disorders (e.g. common vs severe mental disorders). Case studies could examine the nexus between prioritization at the national, regional and global levels. Finally, studies could investigate the role and power of different actor groups during the prioritization process.

COVID-19 has exacerbated mental health needs worldwide at an unprecedented scale, yet global mental health continues to attract limited political attention. Prioritization over the next decade will depend on the capacity of the policy community to harness political support. This paper is timely: it provides an analysis of factors that shaped the prioritization of global mental health over the past three decades and points to barriers and opportunities to inform future research and action.

## Supplementary Material

czac046_Supp
