# Supplementary material for: Establishing political priority for global mental health: a qualitative policy analysis
Source: Health Policy Plan. 2022 Jun 13;37(8):1012–24. doi: 10.1093/heapol/czac046 (PMC9384251; doi:10.1093/heapol/czac046)
Supplement: czac046_Supp [file czac046_supp.zip › Political priority for global mental health - Supplement.pdf]

Supplement to: Iemmi V. 2022. Establishing political priority for global mental health: a qualitative policy analysis. *Health Policy and Planning* published online Jun 13. https://doi.org/10.1093/heapol/czac046.

Supplementary Data

Appendix 1: Key websites……………………………………………………………………………………...2
Appendix 2: Participant characteristics………………………………………………………………………4
Appendix 3: Illustrative quotations for themes and sub-themes……………………………………………..5
Appendix 4: Global attention to mental health in 2000, 2010, and 2020…………………………...………………8

Appendix 1: Key websites

	Websites
**Mental Health-specific**	
Dementia Discovery Fund (DDF)	https://svhealthinvestors.com/funds/the-dementia-discovery-fund
Global Business Collaboration for Better Workplace Mental Health	https://betterworkplacemh.com/
Global Mental Health Action Network (GMHAN)	https://unitedgmh.org/global-mental-health-action-network
Global Mental Health Peer Network (GMHPN)	https://www.gmhpn.org/
Inter-Agency Standing Committee (IASC) Reference Group on Mental Health and Psychosocial Support in Emergency Settings	https://interagencystandingcommittee.org/iasc-reference-group-on-mental-health-and-psychosocial-support-in-emergency-settings
International Alliance of Mental Health Research Funders (IAMHRF)	https://iamhrf.org/
Mental Health Innovation Network (MHIN)	https://www.mhinnovation.net/
MHPSS Network	https://mhpss.net/
Movement for Global Mental Health (MGMH)	http://www.globalmentalhealth.org/
National Institute of Mental Health (NIMH)	https://www.nimh.nih.gov/
Speak Your Mind	https://gospeakyourmind.org/
United for Global Mental Health	https://unitedgmh.org/
World Federation for Mental Health (WFMH)	https://wfmh.global/
World Health Organization (WHO) Department of Mental Health and Substance Use	https://www.who.int/teams/mental-health-and-substance-use
World Mental Health Survey Initiative	https://www.hcp.med.harvard.edu/wmh/index.php
World Network of Users and Survivors of Psychiatry (WNUSP)	http://wnusp.net/
World Psychiatry Association (WPA)	https://www.wpanet.org/
**Other**	
Asia-Pacific Economic Cooperation (APEC)	https://www.apec.org/
Carter Center	https://www.cartercenter.org/
Christian Blind Mission (CBM)	https://www.cbm.org/
Dutch Minister for Foreign Trade and Development Cooperation	https://www.government.nl/ministries/ministry-of-foreign-affairs
Global Alliance for Chronic Diseases (GACD)	https://www.gacd.org/
Global Fund to Fight AIDS, Tuberculosis and Malaria	https://www.theglobalfund.org/en/
Grand Challenges Canada	https://www.grandchallenges.ca/
High-level Political Forum on Sustainable Development	https://sustainabledevelopment.un.org/hlpf
International Committee of the Red Cross (ICRC)	https://www.icrc.org/en
International Disability Alliance (IDA)	https://www.internationaldisabilityalliance.org/
International Organization for Migration (IOM)	https://www.iom.int/
The Lancet	https://www.thelancet.com/
Office of the United Nations High Commissioner for Human Rights (OHCHR)	https://www.ohchr.org/EN/pages/home.aspx
Open Society Foundations (OSF)	https://www.opensocietyfoundations.org/
Organisation for Economic Co-operation and Development (OECD)	https://www.oecd.org/about/
United Kingdom Department for International Development – DFID (now Foreign, Commonwealth & Development Office, FCDO)	https://www.gov.uk/government/organisations/foreign-commonwealth-development-office
United Nations (UN)	https://www.un.org/en/
United Nations Children's Fund (UNICEF)	https://www.unicef.org/
United Nations Department of Economic and Social Affairs (UNDESA)	https://www.un.org/en/desa

	Websites
United Nations High Commissioner for Refugees (UNHCR)	https://www.unhcr.org/uk/
United Nations Human Rights Council (UNHRC)	https://www.ohchr.org/EN/pages/home.aspx
United Nations Inter-Agency Task Force on Non-communicable Diseases (UNIATF)	https://www.who.int/groups/un-inter-agency-task-force-on-NCDs
United Nations Office for the Coordination of Humanitarian Affairs (UNOCHA)	https://www.unocha.org/
United Nations Office on Drugs and Crime (UNODC)	https://www.unodc.org/
United Nations Population Fund (UNPF)	https://www.unfpa.org/
United States Agency for International Development (USAID)	https://www.usaid.gov/
Wellcome Trust	https://wellcome.org/
World Bank Group	https://www.worldbank.org/en/home
World Economic Forum (WEF)	https://www.weforum.org/
World Health Organization (WHO)	https://www.who.int/

Last accessed: 21 December 2021.

Appendix 2: Participant characteristics

	Number of participants (%)	Interviews
**Sex**		
Female	18 (51%)	1–3,5,7,9,12,17,18,25,27–31,33–35
Male	17 (49%)	4,6,8,10,11,13–16,19,20–24,26,32
**WHO Regions**[a]		
African Region	2 (6%)	7,16
Region of the Americas	11 (31%)	6,15,17, 21,25,27–29,32,34,35
European Region	22 (63%)	1–5,8–14,18–20,22–24,26,30,31,33
**Sectors**		
Public sector	15 (43%)	5,6,7,11,13,15,18,24,26,27–29,31,33,34
Private sector	7 (20%)	1,2,5,9,21,23,32
Third sector	11 (31%)	3,8,10,12,16,17,19,22,25,30,35
Multisector partnerships	2 (6%)	14,20

[a] Twelve countries were represented: two in the African region, two in the region of the Americas, and eight in the European region.

Appendix 3: Illustrative quotations for themes and sub-themes

THEME	SUB-THEME	EXAMPLE QUOTATIONS
**Actor power**	Policy community cohesion	'There are a lot more players in the last 10 years, certainly in the last five years.' (interview 16)<br><br>'I think that within the mental health space there is a need for unity, that we need for the various partnerships to come together and work together.' (interview 23)<br><br>'There are *so many* different actors interested (in mental health) and their interests sometimes compete with each other: family members, consumers, psychiatrists, psychologists…' (interview 32)<br><br>'There is the great claim of anti-global mental health people, that global mental health is a front for pharma and a sort of a secretive way of marketing their drugs across the world.' (interview 8)
	Leadership	'Researchers are so dominant in the global mental health field... But they have broadly made the mistake of thinking that science is enough. The science is a *necessary* component, but it's not sufficient.' (interview 19)<br><br>'Mental health is being discussed at the highest level. The Secretary-General of the UN (=United Nations) is pushing…. for UN agencies to engage in mental health.' (interview 28)<br><br>'We're hearing more voices from world leaders like Theresa May and Angela Merkel and Justin Trudeau, and also from people like Prince Harry and Lady Gaga.' (interview 21)
	Guiding institutions	'The world has a debt with Madam Gro Harlem Brundtland. She was the first Director-General in the history of WHO who gave mental health an important visibility… This was absolutely unprecedented.' (interview 22)<br><br>'They (=World Federation for Mental Health) have a network of advocacy groups around the world but unfortunately (they) don't play the role they could.' (interview 32)
	Civil society mobilisation	'One of the big things that is missing is a strong user movement.' (interview 1)<br><br>'The key thing is: just like with HIV/AIDS it wasn't the decision makers who changed the algorithm for whether to invest in HIV/AIDS, it was the patients and the advocates who were affected by it speaking out and *demanding* change.' (interview 21)
**Ideas**	Internal frame	'The Lancet Commission on global mental health (and sustainable development) put very clearly that mental health is not one condition and that it's something that can come all through the life of anyone anywhere, and it applies to all of us independently from whether you live in a high-income country or a low-income country, or (your) gender, age, and so on.' (interview 28)<br><br>'(Addressing) mental illness is not just about medical interventions. It is also a matter of long-term rehabilitation, which means that you have to think about housing, you have to think about economic advancements, livelihoods, and so on and so forth.' (interview 10)

THEME	SUB-THEME	EXAMPLE QUOTATIONS
	External frame	‘There are different messages going out and I think they need to align a bit more and to really ensure that they don’t keep going in different directions.’ (interview 23)<br><br>‘We constructed a sense that this is, if not a growing issue, an issue that is growing to be perceived as important or overdue or needing attention now.’ (interview 8)<br><br>‘…people being in an institution, people tortured, people being in chains. Those stories horrify people, so they do react, and they do want to do something about it.’ (interview 3)<br><br>‘Economic arguments have being paid more attention in the last few years.’ (interview 4)<br><br>‘The most effective argument… has been to show people… how integrating mental health and psychosocial support can improve what they’re already doing, and also then the flipside is: if you *don’t* address mental health and psychosocial support it can really serve as a bottleneck to the outcome that you’re trying to achieve.’ (interview 29)<br><br>‘Mental health for many decades, if not centuries, has been a taboo subject. The stigma and discrimination have been part of the issue.’ (interview 15)<br><br>‘It (=mental health) is coming out of the shadows. People are talking about their suffering and people start realising that the problem is much bigger than previously thought.’ (interview 26)
**Political contexts**	Policy windows	‘Overall, there is globally much more interest in mental health, without a doubt.’ (interview 26)<br><br>‘There are many global as well as regional fora in which mental health has been talked about. And that has been extremely helpful, because they provide the kind of background information and (they) increase the political commitment of people at the highest level.’ (interview 4)<br><br>‘The SDGs (=Sustainable Development Goals) *couldn’t* not have mental health, because society has changed… I think that if we had a fantastic campaign before the MDGs (=Millennium Development Goals), we probably still wouldn’t have done very well. Because the global discourse wasn’t mature, whereas it is now.’ (interview 19)<br><br>‘The (UK) Prime Minister (David) Cameron under the G8 launched the Global Dementia Initiative to try to do something about Alzheimer’s (Disease) and… that has been very successful…. the *attention* to the social impact of the growing burden of dementia and some of the things that can be done about that has really transformed that space.’ (interview 21)
	Global governance structure	‘It (=Convention on the Rights of Persons with Disabilities, CRPD) is one of the strongest of any UN conventions on rights, but it doesn’t count much beyond public shaming.’ (interview 19)<br><br>‘Mental health being part of the Mental Health Action Plan (2013–2020) at WHO… has meant a lot: there is at least on paper a very high-level political commitment.’ (interview 4)

THEME	SUB-THEME	EXAMPLE QUOTATIONS
**Issue characteristics**	Credible indicators	‘Health indicators were and still are quite rare. The predominance is of process indicators.’ (interview 22)<br><br>‘The ability for someone to get better in mental health for a period (of time) and then do worst again, and then get better… (makes) hard to understand… at what point out from an intervention you would say: ‘This person is definitely better, definitely not better.’ (interview 17)
	Severity	‘I think it is also a growing awareness among policy makers… of demographic and epidemiological transitions and the growing importance of noncommunicable diseases and mental health.’ (interview 16)<br><br>‘I think the situation now of the epidemiological data is much better that in the end of nineties, in the beginning of the 2000s.’ (interview 22)
	Effective interventions	‘The early intervention (trials) in the early 2000s… have definitely been a big game changer because they showed it was possible (for interventions to be effective) in low-resource settings.’ (interview 16)<br><br>‘We don’t have some knock-out interventions, like: give us the money and we’ll vaccinate kids and they won’t get the disease for the rest of their life. It’s not as simple as that.’ (interview 11)

Ellipses indicate removed text to shorten quotes, while preserving meaning. Parentheses contain text added by the author to facilitate comprehension. Italics reproduce participants’ emphases. HIV/AIDS, human immunodeficiency virus infection and acquired immunodeficiency syndrome; MDGs, Millennium Development Goals; SDGs, Sustainable Development Goals; UK, United Kingdom; UN, United Nations; WHO, World Health Organization.

Appendix 4: Global attention to mental health in 2000, 2010, and 2020

	2000	2010	2020
**Actor power**			
Policy community cohesion	Fragmented policy community: few actors with different interests and approaches (eg, private sector, radical critics of biomedical psychiatry)	Persistent fragmentation of the policy community; increasing actors and approaches (eg, disability community); attempt to strengthen cohesion by actors (eg, MGMH)	Persistent fragmentation of the policy community and actors increase; attempt to strengthen cohesion by new actors (eg, GMHAN; UGMH)
Leadership	Few strong champions, mainly from academia; very few high-level leaders and champions (eg, US First Lady Rosalynn Carter) promoting the issue	Increasing, yet limited, number of strong champions: new influential individuals, mainly from academia; few supportive high-level leaders (eg, WHO Director-General Gro Harlem Brundtland)	Increasing, yet limited, number of strong champions: growing number of individuals from academia and leaders in international organisations (eg, UN Secretary-General António Guterres, WHO Director-General Tedros Adhanom Ghebreyesus) and national governments (eg, UK Prime Minister David Cameron), and celebrities (eg, Lady Gaga)
Guiding institutions	Absence of a single guiding institution or coordination mechanism, but multiple prominent institutions: WHO, non-governmental organisations (eg, WFMH), and lived experience groups (eg, WNUSP)	Strengthening role of WHO	Persistent strengthening of WHO role; new prominent non-governmental organisations (eg, UGMH) and lived experience groups (eg, GMHPN)
Civil society mobilisation	Few civil society organisations pressing national and international political authorities; establishment of a global network of organisations and people with lived experience (WNUSP)	Increasing, yet limited, civil society organisations; establishment of a movement of individuals and organisations working in global mental health (MGMH)	Persistent increase in number of civil society organisations, yet limited compared to other issues; increasing support from organisations promoting other issues integrating mental health components; new global networks of individuals and organisations working in global mental health (eg, GMHAN, GMHPN)
**Ideas**			
Internal frame	No consensus on definitions of, causes of, and solutions to address mental disorders; two prominent explanatory models (biomedical and cultural); standalone solutions, often restricted to healthcare	Persisting absence of consensus; two prominent explanatory models (bio-psycho-social and human rights); widened standalone solutions and emerging integrated interventions	Progressive convergence towards a consensus in definition, causes, and solutions; increasing importance of integrated solutions across sectors and over the lifetime
External frame	Divided public portrayal; multiple arguments for action often adopted simultaneously (public health, human rights, moral duty); stigma	Persistent divided public portrayal; additional argument for action (economic imperative); decreasing stigma	Persistent divided public portrayal; additional arguments for action (development issue, cost-effective investment, major cause of unhappiness); continued decrease in stigma

	2000	2010	2020
**Political contexts**			
Policy windows 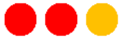	Missed opportunity to link the issue to the MDGs; few high-level meetings on or including mental health; failure to capitalise on humanitarian emergencies	Persistent paucity of high-level meetings; capitalisation upon the 2004 tsunamis in Banda Aceh, Indonesia and Sri Lanka	Successful inclusion of mental health in the SDGs; growing high-level meetings on mental health and on other issues where mental health has increasingly been integrated; COVID-19 pandemic bringing attention to the issue worldwide; increasing (yet limited) mental health activities at national and regional levels
Global governance structure 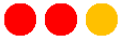	Limited global governance structure: few international legislations, and no global frameworks and plans; unfavourable norms (eg, benefitting vertical issues); few institutions supporting collective action	Emerging global governance structure: an international treaty (CRPD); increasing institutions and coordinating groups supporting collective action (eg, IASC reference group on MHPSS in emergency settings)	Strengthening global governance structure: two global frameworks (SDGs, Sendai Framework) and a global plan (MHAP); more favourable norms (eg, benefitting horizontal issues); additional institutions and coordinating groups supporting collective action (eg, IAMHRF)
**Issue characteristics**			
Credible indicators 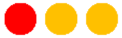	Few credible indicators at both individual and population levels	Increasing, yet limited, credible indicators at population and system levels	Persistent increase in credible indicators at population and system levels, yet limited compared to other issues; inclusion amongst SDGs indicators
Severity 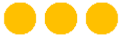	Substantial burden of mental disorders relative to other conditions; paucity of robust and objective data, especially in LMICs; reliance on estimates for global figures	Increasing burden of disease; growth in population-level data, yet limited, especially in LMICs	Persistent increase in burden of disease; sustained growth in population-level data, yet limited compared to other issues
Effective interventions 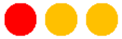	Paucity of simple interventions due to issue characteristics; little evidence on effective and cost-effective interventions, mainly limited to HICs	Increasing evidence, including clinical trials in LMICs	Sustained increase in evidence on effective and cost-effective interventions; little evidence on implementation and scalability in different settings, especially in LMICs; misperception of the issue as a novel field

Three circles illustrate favourability of each determinant of political priority for mental health in 2000, 2010, 2020, respectively. Favourability corresponds to the number of barriers to each determinant. Red, many; Orange, few; Green, none.

CRPD, Convention on the Rights of Persons with Disabilities; GMHAN, Global Mental Health Action Network; GMHPN, Global Mental health Peer Network; HICs, high-income countries; IAMHRF, International Alliance of Mental Health Research Funders; IASC, Inter-Agency Standing Committee; LMICs, low- and middle-income countries; MDGs, Millennium Development Goals; MGMH, Movement for Global Mental Health; MHAP, Mental Health Action Plan; MHPSS, mental health and psychosocial support; SDGs, Sustainable Development Goals; Sendai Framework, Sendai Framework for Disaster Risk Reduction 2015–2030; UGMH, United for Global Mental Health; UK, United Kingdom; UN, United Nations; US, United States; WFMH, World Federation for Mental Health; WHO, World Health Organization; WNUSP, World Network of Users and Survivors of Psychiatry.
